# From fixed activities to personalized treatments in radionuclide therapy: lost in translation?

**DOI:** 10.1007/s00259-017-3859-1

**Published:** 2017-10-27

**Authors:** G. D. Flux, K. Sjogreen Gleisner, C. Chiesa, M. Lassmann, N. Chouin, J. Gear, M. Bardiès, S. Walrand, K. Bacher, U. Eberlein, M. Ljungberg, L. Strigari, E. Visser, M. W. Konijnenberg

**Affiliations:** 1 Joint Department of Physics, Royal Marsden Hospital & Institute of Cancer Research, Downs Road, Sutton, Surrey UK; 2 Department of Medical Radiation Physics, Lund University, Lund, Sweden; 3 Nuclear Medicine, Foundation IRCCS Istituto Nazionale Tumori, Milan, Italy; 4 Department of Nuclear Medicine, University of Würzburg, Würzburg, Germany; 5 Nantes-Angers Cancer Research Center CRCINA, University of Nantes, INSERM UMR1232, CNRS-ERL6001, Nantes, France; 6 Oniris, AMaROC Unit, Nantes, France; 7 Centre de Recherches en Cancérologie de Toulouse, UMR 1037 INSERM Université Paul Sabatier, Toulouse, France; 8 Nuclear Medicine, Molecular Imaging, Radiotherapy and Oncology Unit (MIRO), IECR, Université Catholique de Louvain, Brussels, Belgium; 9 Department of Basic Medical Sciences, Division of Medical Physics, Ghent University, Ghent, Belgium; 10 Laboratory of Medical Physics and Expert Systems, National Cancer Institute Regina Elena, Rome, Italy; 11 Department of Radiology and Nuclear Medicine, Radboud University Medical Centre (RadboudUMC), Nijmegen, The Netherlands; 12 Department of Radiology and Nuclear Medicine, Erasmus MC, Rotterdam, The Netherlands

We read with interest the letter by Giammarile et al. [1] addressing our editorial in which we proposed that the European Medicines Agency should allow the option of a dosimetry-based approach to the treatment of cancer with radionuclide therapy [2]. Our editorial was intended to draw attention to the potential legal issues of recommending an approach to treatment that could contravene the European Council Directive 2013/59 [3] and national legislation, as the directive (article 56) states that “For all medical exposure of patients for radiotherapeutic^1^ purposes, exposures of target volumes shall be individually planned and their delivery appropriately verified...”. This directive is intended to “lay down basic safety standards for the protection of dangers arising from exposure to ionizing radiation” and emphasizes the need for ‘justification’ and ‘optimization’ of intentional radiation exposures of patients.

We do not agree with the conclusion of this letter that cancer therapy with radiopharmaceuticals should be developed “in a similar manner to chemotherapeutics”, “independent of tumor load and metastases” and is “better characterized as a tumor-selective treatment modality with more similarities to systemic chemotherapy”.

In any scientific field concerned with biological effects of radiation, whether for therapy or radiation protection purposes, the effects of radiation on tissue are primarily dependent on the well-established measure *absorbed dose*. Consequently, great efforts are made to calculate absorbed doses in cells, tissues, and organs. The hypothesis that the level of activity administered has a greater impact on treatment outcome than the subsequent biodistribution, the radiation delivery and the absorbed dose is ignoring the results of decades of radiation research on biological systems.

Still, today, prescriptions in radionuclide therapy are most commonly based on a fixed amount of activity for all patients, sometimes tailored to patient weight or body surface area. While this enables therapy to be performed with minimal resourcing or planning, we contend that the development of personalized prescription alternatives based on dosimetry are likely to improve the outcome and cost-benefit of radionuclide therapies. There is increasing evidence that treatment outcome correlates with the absorbed doses delivered to tumors and to healthy organs [4]. For example, in a recent multivariate analysis on radioembolization in hepatocellular carcinoma, lesion absorbed dose was the only factor associated with prolonged overall survival [5]. We agree with Giammarile et al. that it is unfortunate that there have been no randomized controlled trials to evaluate the respective merits of dosimetry-based versus fixed-activity approaches. This need has long been acknowledged [6] and we certainly encourage and will support any initiatives in this area. However, the absence of trials cannot be regarded as a valid reason to hinder the development of dosimetry-based prescriptions and does not justify the avoidance of post-treatment verification of the absorbed doses delivered, which provides patient-specific information without changing patient management. Progress in medicine necessarily entails the introduction of new concepts for which there is a hypothesis of clinical benefit, so that evidence can then be obtained to support or refute such hypotheses. The emergence of PET and PET/CT were strongly supported and endorsed by nuclear medicine before clinical evidence became available.

Undoubtedly, the implementation of dosimetry-based treatment planning and the verification of the absorbed doses delivered following administrations will entail a major reappraisal of the way in which these treatments are conventionally handled. As Giammarile et al. highlighted, there are a number of challenges to be addressed. These include resourcing and training, and possibly new ideas on how to organize the treatment. Such challenges are by no means unique for radionuclide therapy, and should be considered within the perspective of the introduction and evaluation of new techniques in other radiotherapy fields, building on fruitful collaborations between different medical specialties in nuclear medicine, oncology, and medical physics. Training and professional development is necessary and available, not least via the EANM ESMIT teaching initiatives as well as the DoMore track of the EANM annual congress.

A number of justifications are frequently presented to avoid dosimetry. While an absorbed dose calculation is subject to uncertainties that stem from the methodology chain that covers activity determination to the absorbed dose calculation, there is abundant evidence that simplistic prescription methods, based on fixed activities, deliver a range of absorbed doses to normal organs and tumors that are far greater than methodological uncertainties (see e.g., [7–16]).

The role of radiobiology to examine the impact of radioresistance, low and continuous absorbed dose rates, and heterogeneity of uptake at either a cellular, microscopic or macroscopic scale is under investigation, and will expand if dosimetry data are made available to compare with outcomes. We believe that this research field should be encouraged to fully develop the theragnostic advantage that radionuclide therapy can offer.

It is sometimes claimed that the cost of dosimetry is prohibitive. While this may be an issue for established inexpensive treatments, the same cannot be said of the large number of emerging radiopharmaceuticals, for which the cost of extra scans and physics time is minimal in comparison with the cost of the drug itself. It is incongruous to invest heavily in the development of a new drug, but then to forego clinical optimization of that drug. Health economic studies are warranted, whereby the cost of dosimetry may be measured against the potential cost savings of more individualized and optimized treatments.

We endorse the Basic Safety Standards directive of the European Union [3], which states that radiotherapeutic procedures should be both planned and verified. Physicians, medical physicists, and all disciplines within nuclear medicine should jointly promote any efforts that lead to best patient care and that may minimize avoidable short- and long-term toxicity. We foresee a bright future for radionuclide therapy in which dosimetry-based personalized treatments are developed by multidisciplinary engagement, and look forward to continuing discussions on how this should be translated into clinical practice.

## Footnotes

1 According to the Directive (article 4, definition (81)) “radiotherapeutic” means pertaining to radiotherapy, including nuclear medicine for therapeutic purposes”
